# 

**Published:** 1850-01

**Authors:** 


### TO SUBSCRIBERS.

☞ With this number we send our delinquent subscribers their bills, and hope it will not be necessary to insist further upon payment. They are small items to you severally, gentlemen, but without them, to say nothing of the justice of their being paid, it is impossible for us to sustain the Journal.

Our subscribers will do well too, to remember that our terms are three dollars for the current year, unless paid for before the close of the volume, and the next will be the last number.

### NOTICE TO READERS AND CORRESPONDENTS.

We have received Original Communications from Drs. E. S. Cooper, J. S. Jones, David Hutchinson, and R. A. Spencer.

We have received from Messrs. Wood of New York, through J. Keen Jr., & Bro., of Chicago, Thompsons Conspectus of the Pharmacopœias.

From Messrs. Lea & Blanchard, through S. C. Griggs & Co., of Chicago, West on Diseases of Children.

Life of Bishop Quarter—By J. E. McGirr, M. D. (From the Author.)

Also, a number of Introductory Lectures, which our space will not allow us to notice in this number. We will endeavor to pay them our compliments hereafter.

Also, Report of the Indiana Hospital for the Insane. (From Dr. Patterson, Superintendent.)

Miss Dix's Memorial to the Legislature of Alabama, soliciting an appropriation for a Hospital for the Insane: (From the Authoress.)

Also, our usual list of Exchanges.

---

### LIST OF RECEIPTS FOR NOVEMBER & DECEMBER, 1849.

| | |
|---|---|
| **ILLINOIS.** | |
| Drs. Torry, | $2 50 |
| W. P. Richardson, | 2 00 |
| W. H. Davis, | 2 00 |
| J. N. Banks, | 2 00 |
| C. J. Hull, | 2 50 |
| Thomas Stanton, | 2 00 |
| A. Cass, | 4 00 |
| Milton Bacon, | 4 00 |
| W. Henry Davis, | 2 00 |
| E. G. Mygatt, | 4 00 |
| A. W. Rawson, | 2 00 |
| W. F. Colman, | 2 00 |
| C. Goodbrake, | 2 00 |
| P. Reimbold, | 2 00 |
| A. Comstock, | 6 00 |
| E. McArthur, | 2 00 |
| **INDIANA.** | |
| Drs. S. W. Purviance, | 2 00 |
| Refenbarick & Spencer, | 2 00 |
| C. H. Butler, | 2 00 |
| J. S. Baxter, | 2 00 |
| T. W. Meller, | 2 00 |
| R. J. Simpkins, | 4 00 |
| Drs. E. Allen, | $2 00 |
| G. W. Parks, | 2 00 |
| W. Matthews, | 2 00 |
| J. C. Helm, | 2 00 |
| Lot Edwards, | 4 00 |
| W. H. Gifford, | 2 00 |
| Julius Huntington, | 2 00 |
| Joel E. Hendricks, | 2 00 |
| **MICHIGAN.** | |
| Drs. Z. T. Slater, | 1 00 |
| M. B. Burs, | 3 00 |
| J. Higby, | 3 00 |
| S. Stoddard, | 2 00 |
| J. Cornell, | 2 00 |
| **WISCONSIN.** | |
| Drs. C. B. Lake, | 2 00 |
| J. R. Goodno, | 2 00 |
| G. W. Lee, | 3 00 |
| J. H. Turner, | 2 00 |
| **IOWA.** | |
| Drs. J. M. Weatherwax, | 3 00 |
| A. J. Lockhart, | 2 00 |
| Clarke & Bird, | 2 00 |
| A. A. Hemenway, | 4 00 |

